# Macrophage Iron Metabolism Mediates Immunometabolic Reprogramming and Tissue Homeostasis: From Molecular Mechanisms to Clinical Translation

**DOI:** 10.3390/cells15100895

**Published:** 2026-05-14

**Authors:** Mingwei Wang, Qiaohui Ying, Qing Li, Xia Lou, Shuchang Dai, Zhong Liu

**Affiliations:** 1 Institute of Blood Transfusion, Chinese Academy of Medical Sciences and Peking Union Medical College, Chengdu 610052, China; sxswangmingwei@student.pumc.edu.cn (M.W.); liqing1026370@163.com (Q.L.); lx750780@163.com (X.L.); dschang2024@163.com (S.D.); 2 Key Laboratory of Transfusion Adverse Reactions, Chinese Academy of Medical Sciences, Chengdu 610052, China; 3 Center of Craniofacial Orthodontics, Department of Oral and Cranio-Maxillofacial Surgery, Shanghai Ninth People’s Hospital, Shanghai Jiao Tong University School of Medicine, College of Stomatology, Shanghai Jiao Tong University, National Center for Stomatology, National Clinical Research Center for Oral Disease, Shanghai Key Laboratory of Stomatology, Shanghai 200011, China; a619289369@outlook.com

**Keywords:** CD163, mechanotransduction, epigenetic remodeling, tumor-associated macrophages, immunometabolism, nanomedicine

## Abstract

Background: Macrophages were long regarded as passive executors of erythrophagocytosis responsible for systemic iron recycling. However, increasing evidence has reframed them as immunometabolic hubs that sense diverse environmental cues to modulate systemic iron homeostasis. Main body: This review examines the molecular architecture underlying macrophage iron metabolism and outlines how iron metabolic processes are dynamically regulated across spatial and temporal scales through the integration of mechanotransductive, mitochondrial, and epigenetic signaling pathways. Across disease contexts, macrophage iron handling displays marked heterogeneity, exemplified by contact-dependent iron transfer in tumors and ferroptosis-driven instability in cardiovascular disease. In cardiovascular pathologies, iron overload is associated with enhanced ferroptosis-related cascades that contribute to atherosclerotic plaque instability. Furthermore, at mucosal interfaces, host–pathogen competition over nutritional immunity highlights epigenetic strategies by which pathogens perturb host iron machinery. Conclusions: Linking these mechanistic insights to clinical translation, emerging therapeutic strategies are discussed that move beyond non-specific systemic iron chelation toward more targeted interventions. These include engineering macrophages for targeted drug delivery, exploiting nanomedicine-based redox modulation to influence macrophage phenotypes, and non-invasive regulation via the gut microbiota–epigenetic axis. Collectively, elucidating macrophage iron metabolic networks provides a conceptual framework for the development of precision approaches to inflammatory, metabolic, and malignant diseases.

## 1. Introduction

As one of the most abundant transition metals on Earth, iron is fundamentally critical for the evolution of life [1,2]. Its distinctive redox characteristics render it an indispensable cofactor for fundamental biological processes, including DNA synthesis, oxygen transport, electron transfer within the mitochondrial respiratory chain, and numerous enzymatic reactions [3,4,5]. However, this potent chemical activity represents a double-edged sword. Cellular redox-active iron is not truly free, but is largely maintained within the labile iron pool (LIP), where it is loosely associated with low-molecular-weight ligands such as glutathione or citrate [6,7]. The LIP is constrained by cellular buffering mechanisms and regulated in part by the iron-responsive element/iron regulatory protein (IRE/IRP) system, which coordinates iron uptake, storage, and export [8,9]. It is only when this intricate homeostatic machinery is overwhelmed or genetically disrupted that excess labile iron catalyzes the formation of highly destructive reactive oxygen species (ROS) via the Fenton reaction, precipitating lipid peroxidation, protein damage, and ultimately cell death [10,11,12]. Consequently, mammals have evolved intricate and sophisticated regulatory systems to maintain systemic iron homeostasis, with macrophages serving as the central hub of this critical physiological process.

Historically, macrophages were characterized merely as “scavengers” or immune sentinels dedicated to pathogen clearance and debris removal. However, the past decade has witnessed a paradigm shift in our understanding of these versatile cells. Beyond simply processing iron, macrophages orchestrate a complex iron flux by integrating diverse mechanisms for acquisition, storage and efflux [13,14]. Crucially, iron has transcended its classical role as a passive metabolic substrate to emerge as a pivotal signaling molecule determining cell fate. Intracellular iron availability dictates metabolic reprogramming, epigenetic landscapes, and phenotypic plasticity (Box 1).

Box 1 **Key Regulatory Layers of Macrophage Iron Metabolism.**

- **Mechanotransduction:** Hemodynamic forces are sensed by ion channels (e.g., PIEZO1) to translate physical cues into systemic iron mobilization signals.
- **Mitochondrial Metabolism:** Intracellular iron directly dictates bioenergetic transitions (glycolysis vs. OXPHOS) and cell fate (survival vs. ferroptosis).
- **Epigenetic Remodeling:** Iron ions act as essential cofactors for demethylases, directly linking cellular iron pools to global chromatin accessibility and gene expression.
- **Disease Hijacking:** Pathological microenvironments (e.g., tumors, atherosclerotic plaques) subvert physiological iron-handling machinery to support malignant growth or amplify inflammatory tissue injury.

## 2. The Molecular Logic of Macrophage Iron Handling

**Figure 1:**
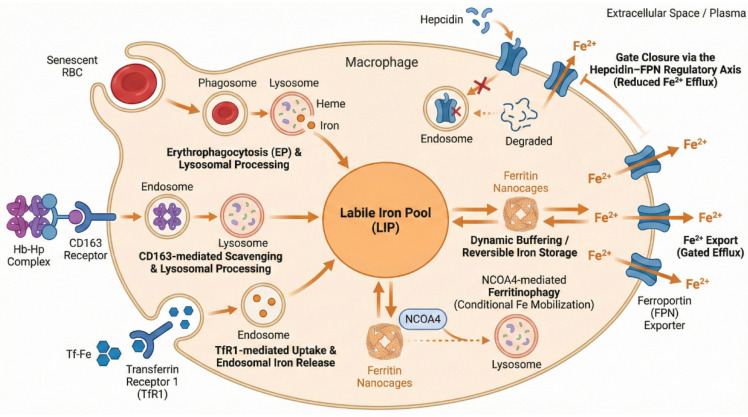
Core molecular architecture of macrophage iron handling: Macrophages acquire iron through erythrophagocytosis, CD163-mediated hemoglobin scavenging, and TfR1-dependent uptake of transferrin-bound iron. These inputs converge on the labile iron pool (LIP), which is buffered by ferritin storage, mobilized through NCOA4-dependent ferritinophagy, and exported through ferroportin (FPN). Hepcidin restricts iron export by inducing FPN internalization and degradation.

As the central hub of systemic iron recycling, macrophages orchestrate a multifaceted metabolic program that precisely balances iron acquisition, storage, and mobilization. Classically, this system relies on a diverse array of input interfaces—ranging from erythrophagocytosis (EP) and CD163-mediated hemoglobin scavenging to transferrin receptor 1 (TfR1) uptake—which collectively fuel the labile iron pool (LIP). Intracellularly, this flux is dynamically buffered by the NCOA4-ferritin axis and gated by the sole iron exporter, ferroportin (FPN), under the systemic governance of the hepcidin-FPN axis (Figure 1) [15,16,17,18]. While the biochemical identities of these players are well established, recent advances highlight that iron-handling machinery is a highly plastic architecture regulated by structural conformations, autocrine signaling, and niche-specific kinetics. In this section, we dissect the structural basis and micro-environmental logic that define the macrophage’s capacity to manage iron.

### 2.1. Structural Basis of CD163-Mediated Hemoglobin Scavenging

**Figure 2:**
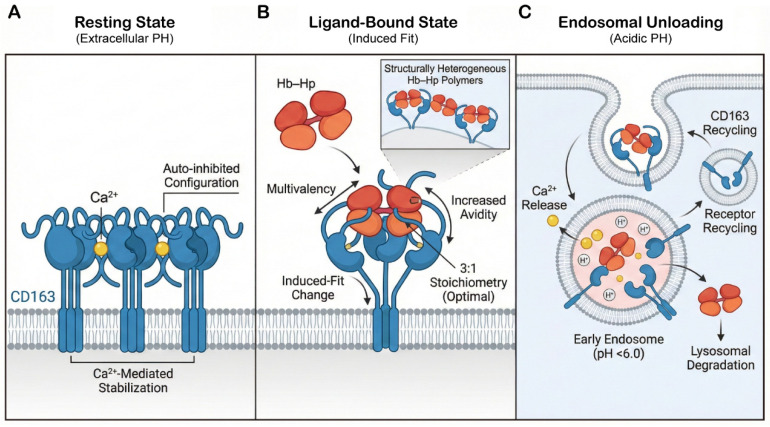
Structural basis of CD163-mediated hemoglobin scavenging during hemolytic stress: (**A**) In the resting state, CD163 forms auto-inhibited, calcium-stabilized multimers. Upon ligand encounter, it undergoes induced-fit rearrangement to form multivalent interactions with Hb–Hp complexes (optimal 3:1 stoichiometry). Acidic endosomal pH then disrupts Ca^2+^-coordination, releasing ligands for lysosomal degradation and allowing receptor recycling. (**B**) Membrane-associated cues induce mechanosensitive calcium influx to fine-tune intracellular iron processing. (**C**) Autocrine purinergic signaling provides a regulatory brake on iron mobilization, stabilizing intracellular availability.

Quantitatively, the vast majority of steady-state macrophage iron recycling is driven by erythrophagocytosis (EP), which clears intact senescent erythrocytes, alongside TfR-dependent internalization for basal cellular needs. However, under specific conditions of intravascular hemolysis or tissue hemorrhage, the scavenger receptor CD163 emerges as a highly specialized and indispensable molecular clearance system. Rather than being the continuous frontline of daily iron flux, CD163 functions to swiftly sequester toxic cell-free hemoglobin-haptoglobin (Hb-Hp) complexes from the circulation [19,20,21]. Recent cryo-electron microscopy (cryo-EM) studies have revealed that this is not a rigid “lock-and-key” match, but rather a dynamic process of “induced fit” (Figure 2).

In the resting state, the extracellular domain of CD163 assembles into multimers (predominantly trimers) within the membrane-proximal region via calcium-mediated interactions, forming an auto-inhibited configuration. In this state, the “arms” of adjacent protomers associate with one another, causing the ligand-binding surfaces to be sterically masked, thereby effectively preventing non-specific binding [22]. Upon encountering Hp-Hb complexes, the receptor undergoes a dramatic conformational switch. Structural analysis reveals that CD163 is not a static receptor but rather acts like flexible “arms” protruding from a membrane base, capable of “moulding” its binding interface to fit the shape of the ligand. While the classic 3:1 stoichiometry (where three CD163 protomers cooperatively “clamp” a single Hp-Hb dimeric unit) provides the highest binding stability [23], this unique structural flexibility allows CD163 to adapt to and capture stoichiometrically diverse and structurally heterogeneous Hp-Hb polymers. This multivalent architecture is critical for biological function. Monomeric CD163, lacking synergistic effects, cannot efficiently endocytose low-affinity ligands; it is only through multimerization—forming a “molecular net”—that the receptor generates sufficient avidity to capture trace amounts of complexes from the blood [24].

Crucially, the entire assembly functions as a pH-sensitive molecular switch. The binding interface relies heavily on a calcium (Ca^2+^)-coordinated electrostatic network: key acidic residues within the CD163 SRCR domains form tight salt bridges with basic lysine residues on the surface of Hp and Hb, bridged by Ca^2+^ [25]. When the receptor-ligand complex is internalized into the early endosome (pH < 6.0), the acidic microenvironment triggers histidine protonation, disrupting the electrostatic network and inducing Ca^2+^ release. This structural instability forces Hb-Hp to dissociate for lysosomal degradation, while the CD163 receptor recycles back to the cell surface [23]. Thus, the macrophage’s capacity for heme detoxification is intrinsically encoded within the atomic-level interplay of precision and flexibility of the CD163 receptor.

### 2.2. PIEZO1-Dependent Mechanotransduction in Iron Metabolism

While the conformational plasticity of CD163 resolves the issue of “biochemical specificity” in iron acquisition, the macrophage’s sensing of physical cues dictates the “spatiotemporal rate” of iron flux [26]. Historically, models of iron regulation have focused predominantly on soluble biochemical factors, such as circulating iron levels and inflammatory cytokines. However, Atcha H. et al. introduced a novel physical dimension to this paradigm: ion channel-mediated mechanotransduction [27]. Given that macrophages reside in hemodynamically complex niches like the splenic red pulp and hepatic sinusoids, they are perpetually subjected to fluid shear stress and compressive forces.

The mechanosensitive ion channel PIEZO1 has been identified as the core component of this physio-biochemical interface [28,29]. Beyond regulating phagocytic activity and erythrocyte turnover, PIEZO1 functions as a direct quantitative modulator for hepcidin expression. Mechanistically, hemodynamic stimuli activate PIEZO1, triggering a robust influx of cytosolic calcium. This calcium signaling cascade subsequently suppresses the NF-κB pathway, thereby significantly downregulating hepcidin transcription [30]. This suggests that mechanical force acts as a “pro-mobilization signal,” instructing macrophages to accelerate iron recycling and release during periods of high hemodynamic activity (indicative of active hemolysis).

This finding holds profound implications for human genetics and evolution. Approximately one-third of individuals of African descent carry a specific *PIEZO1* gain-of-function allele (E756del), which is significantly associated with elevated plasma ferritin levels [30,31]. This correlation suggests that the transduction of microenvironmental physical properties into systemic iron signaling represents an ancient evolutionary adaptation—likely conferring resistance to anemia or infection historically—but one that has emerged as a genetic risk factor for iron overload in modern populations.

Intriguingly, this PIEZO1-driven calcium influx may offer a synergistic link to the calcium-dependent architecture of CD163 described above. We speculate that mechanical force-induced cytosolic calcium transients act not only as transcriptional messengers but may also promote actin cytoskeletal remodeling and vesicle trafficking, thereby accelerating the recycling of CD163 to the plasma membrane. We hypothesize this “mechano-chemical coupling” would ensure that when macrophages sense high shear stress, they not only genetically unblock iron export (via hepcidin suppression) but also cellularly fortify their scavenging capacity by mobilizing CD163 receptors via calcium signaling. However, this proposed synergy remains theoretical and warrants rigorous experimental validation.

In summary, the PIEZO1 channel acts as a mechanotransductive sensor that translates hemodynamic physical forces into calcium-dependent suppression of hepcidin, thereby accelerating iron mobilization during periods of high physiological demand.

### 2.3. Autocrine Purinergic Regulation of Iron Homeostasis

Beyond processing physical mechanical forces, macrophages must also interpret metabolic danger signals within their microenvironment. A recent study has proposed that the purinergic receptor P2Y12 might act as a biochemical “brake” to promote iron retention [32]. Although P2Y12 has long been characterized as a canonical receptor for platelet aggregation and glial cell function [33,34], its potential involvement in regulating iron metabolism within peripheral tissue macrophages is an emerging concept. Based on observations in zebrafish and murine models, it was suggested that genetic ablation or pharmacological inhibition of P2Y12 results in elevated serum iron levels and depleted iron stores in splenic and hepatic macrophages [32]. The authors of this study proposed a potential ‘extracellular ATP-P2Y12-NF-κB-Hepcidin’ autocrine loop, hypothesizing that local hepcidin production might seal the iron efflux channel during tissue injury. Encouragingly, this emerging concept is beginning to gain independent experimental support; a recent study demonstrated that pharmacological inhibition of P2Y12 (using ticagrelor) modulates ferroptosis-related iron handling and upregulates iron-storage proteins like FTH1, thereby mitigating macrophage-driven inflammation in an experimental rheumatoid arthritis model [35]. However, it is crucial to note that this P2Y12-mediated iron regulation axis is currently supported by limited primary evidence. Extensive independent investigations and confirmation by other authoritative studies are required to fully validate this mechanism and establish its universal physiological relevance.

Upon activation by extracellular ATP or ADP—acting as well-established Damage-Associated Molecular Patterns (DAMPs) released from injured cells [36]—P2Y12 signaling sustains the phosphorylation of the NF-κB p65 subunit. This constitutive signaling activity is required to drive the local transcription of hepcidin, consistent with the classical role of NF-κB in inducing hepcidin expression during inflammation [37]. The locally produced hepcidin subsequently acts in an autocrine manner to trigger the internalization and degradation of FPN, thereby sealing the iron efflux channel.

Physiologically, this mechanism likely represents a localized “nutritional immunity” strategy: at sites of tissue injury, macrophages utilize P2Y12 to rapidly “lock” iron intracellularly, preventing free iron from catalyzing oxidative stress or fueling pathogen growth. Furthermore, this finding has significant translational implications. It suggests that widely used P2Y12 antagonists may inadvertently uncouple this iron retention mechanism, potentially altering systemic iron homeostasis in long-term users—a drug–metabolism interaction that warrants further clinical investigation.

### 2.4. Kinetic Control of Intracellular Iron Processing

Upon traversing the plasma membrane, the processing kinetics of internalized iron are strictly dictated by its physicochemical stability. The fate of internalized iron within the macrophage is not a singular, linear pathway; rather, it is highly contingent upon the stability and particle size of the iron complex. This dependency is most vividly illustrated by the distinct cellular handling of clinical intravenous iron formulations.

Comparative studies between iron sucrose (IS) and ferric carboxymaltose (FCM) have revealed two fundamentally different processing architectures [38]. IS, a complex with lower thermodynamic stability, undergoes rapid disassembly within the acidic lysosomal compartment. This swift degradation results in a “pulse-like” release of ferrous iron. While effective for rapidly replenishing LIP and triggering an immediate upregulation of ferritin, this surge often provokes a transient spike in ROS, activating Nrf2-mediated oxidative stress responses that challenge the cell’s antioxidant reserves [39]. In contrast, the structurally more robust FCM exhibits a distinct delayed kinetic profile. Following uptake, FCM is not immediately degraded but is instead sequestered for prolonged periods within enlarged late endosomes/lysosomes. This phenomenon has been metaphorically described as the “Hamster Effect”, wherein the macrophage “hoards” the stable complex within vesicles—analogous to a hamster storing food—allowing for a slow, sustained release of iron [38]. Mechanistically, this sequestration is strictly governed by the endosomal pH gradient, as inhibition of the vacuolar H^+^-ATPase effectively halts iron mobilization [40]. This “endosomal sequestration” mechanism serves a dual physiological function: it creates an intracellular depot for prolonged systemic iron supply while minimizing Fenton reaction-mediated cytotoxicity. Thus, the macrophage functions not merely as a passive transit pipe, but as a sophisticated intracellular iron buffering and controlled-release system.

### 2.5. Duodenal Macrophages and Local Iron Interception

Moving beyond the intracellular buffering capacity described in the iron buffering and controlled-release system model, macrophages at the strategic portal of dietary iron entry—the duodenum—exhibit a distinct form of architectural adaptation: extracellular interception. While the aforementioned mechanisms focus on handling internalized iron, resident CD68^+^ macrophages in the duodenal lamina propria function as active gatekeepers to prevent iron uptake at the source [41].

Distinct from the systemic regulation by hepatic hepcidin, these macrophages employ a local, non-canonical surveillance mechanism driven by the mTORC1 signaling pathway [42]. Upon activation by bacterial cues or microenvironmental stress, mTORC1 signaling triggers the specific upregulation and secretion of serine proteases into the interstitial space [41]. This secretion leads to the local proteolytic degradation of Transferrin (Tf). Since ferrous iron exported by enterocytes must be rapidly loaded onto interstitial Tf for effective transport into the systemic circulation, macrophage-mediated proteolysis of this essential carrier results in a significant reduction in local transferrin saturation. Effectively, this mechanism severs the transport pathway in the interstitial “no-man’s-land” between the enterocyte and the vasculature. Particularly during intestinal infections, this mechanism induces functional hypoferremia to deny pathogens access to dietary iron, functioning independently of systemic hepcidin levels. These findings complete the panorama of macrophage iron architecture: demonstrating a spectrum of functions from intracellular sequestration to extracellular transport blockade.

## 3. Iron as a Signaling Hub Regulating Macrophage Phenotypic Remodeling

**Figure 3:**
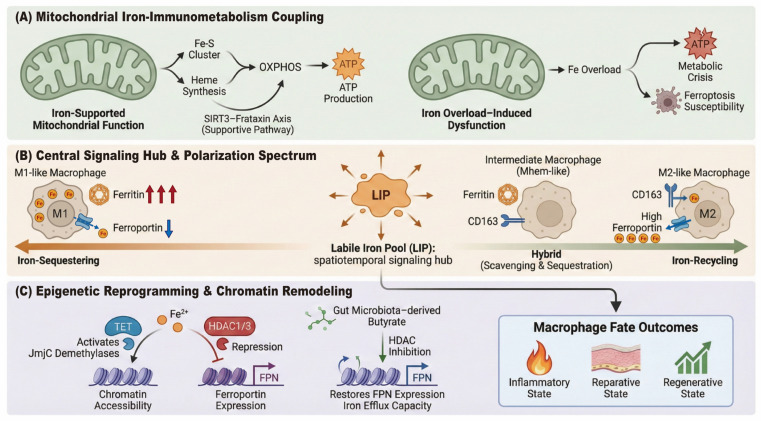
Iron as a signaling hub orchestrating macrophage phenotypic remodeling: (**A**) Metabolic level: Mitochondrial iron sustains OXPHOS via the SIRT3–Frataxin axis, whereas iron overload triggers metabolic crisis and ferroptosis. (**B**) Phenotypic level: Spatiotemporal dynamics of the labile iron pool (LIP) shape a polarization spectrum from iron-sequestering M1-like to iron-recycling M2-like cells, including hybrid Mhem-like states. (**C**) Epigenetic level: Fe^2+^ enables TET/JmjC demethylase-driven chromatin accessibility. Conversely, inflammatory HDAC1/3-mediated ferroportin repression can be relieved by microbiota-derived butyrate.

Iron transcends its classical role as a passive metabolic substrate to establish a central “Signaling Hub” within the macrophage. This regulatory capacity extends far beyond mere nutritional support: the spatiotemporal dynamics of LIP, the bioactive byproducts of heme catabolism, and the assembly integrity of Iron-Sulfur (Fe-S) clusters function as critical checkpoints. These iron-derived signals do not exist in isolation but are functionally integrated with metabolic reprogramming, epigenetic remodeling, and canonical polarization pathways (Figure 3).

### 3.1. Iron-Dependent Macrophage Polarization Across a Phenotypic Spectrum

The classical classification of macrophages exhibits distinct metabolic signatures along the axis of iron handling. M1-like macrophages are characterized by a predominant “iron-sequestering” phenotype. To enforce nutritional immunity, M1 cells robustly upregulate ferritin (FTH1) to sequester iron in a chemically inert form, while simultaneously downregulating FPN and often limiting uptake via the transferrin receptor (TfR1) [43,44]. The net effect of this reprogramming is to restrict iron bioavailability, thereby depriving pathogens of this essential nutrient. Although free iron availability is strictly curtailed, the retained iron remains a requisite cofactor for sustaining the activity of key host defense enzymes, such as inducible nitric oxide synthase (iNOS) [45]. In contrast, M2-like macrophages adopt an “iron-recycling” phenotype. They efficiently scavenge heme-iron via high expression of CD163 and facilitate iron efflux through upregulated FPN [44,46]. This “high-flux” metabolic profile is designed to redistribute iron to the microenvironment, supporting tissue repair, erythropoiesis, and angiogenesis rather than pathogen defense.

However, the relationship between iron status and polarization is bidirectional: iron itself acts as a driver of phenotypic remodeling. For instance, inducing intracellular iron overload and ROS bursts via iron-based nanomaterials can forcibly reprogram pro-tumorigenic M2 macrophages into a tumor-killing M1 state [47]. Conversely, promoting iron efflux (e.g., via hepcidin deficiency) favors the maintenance of an M2-like anti-inflammatory profile [48].

More importantly, recent single-cell transcriptomics challenge this binary boundary, revealing a “phenotypic spectrum” [49]. A prime example is the M_hem_ (hemorrhage-associated) macrophage found in atherosclerotic plaques or areas of tissue hemorrhage [50]. These cells exhibit a hybrid profile: they simultaneously express high levels of the M2 marker CD163 (to scavenge heme) and the M1 marker ferritin (to sequester iron and prevent oxidative damage), yet they lack the classic pro-inflammatory cytokine signature of M1 cells. Such “hybrid” or “intermediate” states demonstrate that macrophages tailor their metabolic programs to the specific iron load of their microenvironment, creating a dynamic functional continuum that transcends rigid M1/M2 definitions.

### 3.2. Mitochondrial Iron as a Determinant of Macrophage Immunometabolism

Macrophage activation is accompanied by profound metabolic transitions. While M1 pro-inflammatory responses rely primarily on glycolysis, the M2 anti-inflammatory and reparative phenotype is sustained by oxidative phosphorylation (OXPHOS) [51,52]. Iron, serving as the obligatory core component of Iron-Sulfur (Fe-S) clusters and heme prosthetic groups within the electron transport chain, constitutes the “catalytic backbone” of this bioenergetic machinery [53,54].

The SIRT3-Frataxin axis has emerged as a critical molecular bridge linking mitochondrial iron metabolism to macrophage immunofunction. SIRT3, a mitochondrial deacetylase, maintains the activity of the iron chaperone Frataxin (FXN) via deacetylation of its lysine residues [55]. The SIRT3–Frataxin axis illustrates how mitochondrial iron handling may regulate macrophage efferocytosis and inflammatory activation. In an angiotensin II (Ang II)-induced cardiac injury model, myeloid SIRT3 deficiency increased FXN K189 acetylation, impaired FXN activity and Fe-S cluster-dependent mitochondrial complex I function, and promoted mitochondrial labile iron accumulation and lipid peroxidation during apoptotic cardiomyocyte efferocytosis [55]. These changes were associated with reduced MerTK expression, defective efferocytosis, and pro-inflammatory macrophage activation. However, as these findings are mainly based on mouse cardiac injury models and in vitro macrophage systems, their relevance to other disease contexts and human macrophages requires further validation.

Conversely, within the pathological microenvironment of atherosclerosis, dysregulated iron metabolism drives plaque instability via a distinct trajectory. In iron-loaded foamy macrophages, the HMOX1-LDHB-TFAM axis has been identified as a novel pathogenic mechanism [56]. While HMOX1 is canonically viewed as cytoprotective, under high-iron conditions, upregulated HMOX1 physically interacts with Lactate Dehydrogenase B (LDHB). This complex recruits and activates the mitochondrial protease LONP1, leading to the aberrant degradation of Mitochondrial Transcription Factor A (TFAM). The loss of TFAM results in the failure of respiratory chain complex assembly and ATP depletion, plunging the macrophage into a “metabolic crisis” that renders it highly susceptible to ferroptosis [57,58]. This metabolic collapse contributes significantly to the formation of the necrotic core within atherosclerotic plaques.

In summary, mitochondrial iron is a critical determinant of macrophage fate; its adequate supply supports OXPHOS-driven M2 polarization via the SIRT3-Frataxin axis, whereas pathological iron overload triggers mitochondrial bioenergetic collapse and ferroptotic death in disease contexts.

### 3.3. Iron–Chromatin Interactions in Epigenetic Remodeling

Parallel to its role in mitochondrial bioenergetics, iron functions as an obligatory cofactor for epigenetic remodeling within the nucleus. This “metabo-epigenetic coupling” is primarily manifested in the direct regulation of demethylase activity. Fe^2+^ is an essential cofactor for the α-ketoglutarate-dependent dioxygenase family, which includes the TET enzymes (responsible for DNA demethylation) and JmjC domain-containing proteins (responsible for histone demethylation) [59,60]. Consequently, fluctuations in LIP are not merely ionic variations but translate directly into global alterations in chromatin accessibility and gene expression landscapes.

Reciprocally, epigenetic machinery exerts hierarchical control over iron metabolism genes. In inflammatory microenvironments, the NF-κB signaling pathway silences FPN via epigenetic repressors to enforce an iron-sequestering phenotype [61]. Specifically, inflammatory stimuli (e.g., TLR ligands) recruit histone deacetylases HDAC1 and HDAC3 to the antioxidant response element within the FPN promoter. The resulting deacetylation induces chromatin compaction, sterically hindering the binding of the transcription factor Nrf2 and leading to transcriptional silencing of FPN. Intriguingly, this mechanism unveils a “microbiota-gut-macrophage axis” regulating iron homeostasis. Butyrate, a short-chain fatty acid metabolite derived from gut microbial fermentation, acts as a potent natural HDAC inhibitor capable of breaking this epigenetic blockade [62]. By preventing HDAC recruitment to the Slc40a1 promoter, butyrate restores histone acetylation and rescues the iron efflux capacity of macrophages [63]. In models of Inflammatory Bowel Disease (IBD), butyrate supplementation has been shown to alleviate macrophage iron overload via this epigenetic resetting mechanism.

## 4. Macrophage Iron Dysregulation in Disease Pathology

**Figure 4:**
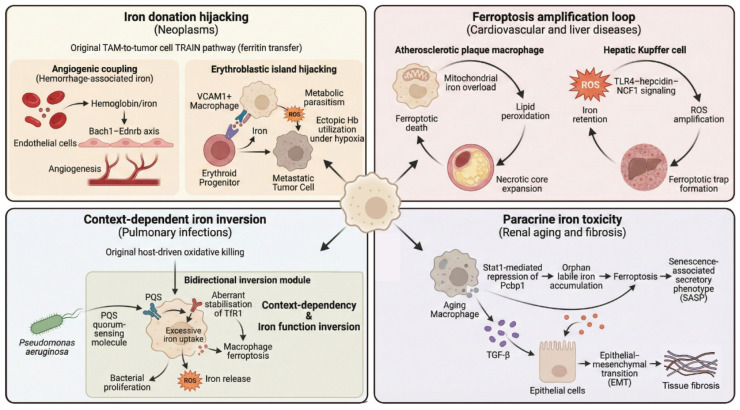
Disease-specific hijacking modes of macrophage iron metabolism: A conserved iron-handling framework is subverted across pathologies. Neoplasms: TAMs are reprogrammed to donate iron via angiogenic coupling, erythroblastic islands, and TRAIN, supporting tumor growth. Cardiovascular & Liver Diseases: Sustained iron overload drives self-amplifying ferroptosis in plaque macrophages and Kupffer cells, exacerbating tissue injury. Pulmonary Infections: Iron functions dually as a host bactericidal effector or a pathogen-exploited trigger for macrophage ferroptosis. Aging Kidneys: Stat1 represses the Pcbp1 chaperone, inducing macrophage senescence and paracrine profibrotic signaling.

The intricate iron-handling machinery of macrophages represents a double-edged sword: indispensable for tissue homeostasis yet intrinsically vulnerable to pathological subversion. When the molecular architecture and signaling hubs discussed above become dysregulated or are “hijacked” by pathological cues—such as the tumor microenvironment or protein aggregates—macrophages transition from physiological guardians to drivers of disease (Figure 4).

### 4.1. Iron Exploitation in the Tumor Microenvironment

In neoplasms, the iron-handling machinery of macrophages is hijacked to satisfy the profound iron addiction of tumor cells to sustain rapid proliferation, and within the Tumor Microenvironment (TME), Tumor-Associated Macrophages (TAMs) are frequently subverted into willing suppliers of this metabolic currency [64,65,66]. First, angiogenic coupling is driven by iron handling. In hemorrhagic tumor regions, specialized iron-handling TAMs (iTAMs) metabolize heme-iron derived from erythrocytes to activate the Bach1-Ednrb axis [67]. This pathway not only detoxifies heme but actively drives pathological angiogenesis, elucidating why iron-rich, hemorrhagic tumor phenotypes correlate with poor prognosis and resistance to immunotherapy. Second, the subversion of the hematopoietic niche highlights the parasitic nature of bone metastases. Here, tumor cells hijack the physiological “Erythroblastic Islands.” Macrophages (specifically VCAM1^+^ subsets) that regulate normal erythropoiesis are reprogrammed to divert iron fluxes away from erythroid progenitors and towards cancer cells [68]. This resource theft allows tumors to ectopically express hemoglobin to survive hypoxia, while simultaneously starving normal hematopoiesis, constituting a key mechanism for refractory anemia in advanced cancer. Finally, contact-dependent transfer (TRAIN) offers a therapeutic window. Beyond soluble iron release, macrophages utilize a sophisticated mechanism termed TRAIN (TRAnsfer of Iron-binding proteiN) to directly “inject” ferritin into tumor cells via cell-to-cell contact [68,69]. While physiologically serving as a lifeline for tumor growth, this pathway represents a fatal vulnerability: it functions as a potential “Trojan Horse” strategy—a mechanism wherein the tumor’s avidity for iron is exploited to covertly deliver cytotoxic payloads inside the cells. Nanotherapeutics designed to hijack this high-efficiency transport route can turn the tumor’s iron avidity into a delivery vector for cytotoxic payloads, perfectly illustrating the “double-edged” potential of targeting macrophage iron metabolism.

### 4.2. Ferroptosis and Iron-Driven Instability in Cardiovascular Disease

In cardiovascular diseases, macrophage iron overload initiates a self-amplifying ferroptosis cascade that destabilizes tissue architecture subject macrophages to a dual burden of erythrocyte lysates and oxidized lipids. This extreme microenvironment positions ferroptosis, rather than classical apoptosis, as the central molecular driver of plaque instability [70,71,72]. Single-cell transcriptomics have specifically highlighted the metabolic vulnerability of the Trem2-low foam cell subset: compromised in their oxidative phosphorylation capacity, these cells fail to buffer the oxidative stress triggered by mitochondrial iron overload, leading to fulminant ferroptosis within the plaque [73]. The release of lipid peroxides from these dying cells propagates a chain reaction of cell death, directly expanding the necrotic core and precipitating fibrous cap rupture and acute thrombosis [56].

Compounding this pathological landscape, pharmacological interventions targeting thrombosis may inadvertently perturb this delicate iron balance. The P2Y12 receptor, a canonical target for antiplatelet therapy, has recently been suggested as a potential regulator of macrophage iron homeostasis. If confirmed by further studies, this implies that while P2Y12 antagonists (e.g., clopidogrel) are effective in preventing thrombosis, their blockade of macrophage signaling might paradoxically disrupt intracellular iron handling, paradoxically exacerbating intra-plaque iron deposition and inflammation under specific conditions [32]. This interaction suggests a need to re-evaluate the immunometabolic sequelae of long-term antiplatelet therapy.

Facing a surge of labile iron in the ischemic myocardium during reperfusion, macrophages secrete extracellular vesicles (EVs) naturally enriched with Transferrin Receptor 1 (TfR1). These vesicles function as roving endogenous “nanosponges,” actively sequestering excess iron from the interstitium. While a recent study proposed that these EVs mitigate ferroptotic stress in cardiomyocytes and preserve residual cardiac function, these findings must be interpreted with caution [74]. The protective efficacy was primarily demonstrated in in vitro models. Furthermore, their in vivo assessment of cardiac iron overload relied heavily on measuring tissue transferrin levels. Given that transferrin is predominantly synthesized in the liver and functions as a circulating carrier rather than an intracellular iron-binding protein, its use as a primary proxy for cardiomyocyte iron load limits the conclusiveness of these in vivo findings. Thus, while the concept of EV-based iron scavenging is highly intriguing, more rigorous in vivo validation using direct intracellular iron quantification is required.

### 4.3. Macrophage Iron Toxicity in Chronic Liver Disease

As the central hub of systemic iron homeostasis, the liver is uniquely vulnerable to macrophage-mediated iron toxicity. In Metabolic Dysfunction-Associated Steatohepatitis (MASH), Kupffer cell-derived Neutrophil Cytosolic Factor 1 (NCF1) acts as a pathological catalyst: it not only generates ROS but also enforces intracellular iron retention via the TLR4-hepcidin axis. This culminates in lethal ferroptosis and the catastrophic depletion of protective resident Kupffer cells, triggering a compensatory infiltration of pro-inflammatory monocyte-derived macrophages [75]. A parallel trajectory occurs in Alcohol-Associated Liver Disease (ALD), where ethanol-induced eryptosis overwhelms Kupffer cells with a heme-iron surge that precipitates ferroptosis [76]. Collectively, these mechanisms underscore a critical therapeutic imperative: moving beyond broad-spectrum chelation toward precision strategies, such as NCF1 inhibition, that specifically preserve resident Kupffer cell viability without disrupting systemic iron homeostasis.

### 4.4. Iron-Dependent Host–Pathogen Interactions in the Lung

At the pulmonary mucosal interface, macrophage iron metabolism operates in a context-dependent manner, oscillating between host defense and pathological inversion to maintain iron homeostasis under extreme metabolic pressure. On this frontline, the role of iron transcends the simplistic dogma of “nutritional immunity” (depriving pathogens of iron), exhibiting profound context-dependency and characterized by a dynamic and antagonistic interplay between host defense and bacterial evasion.

This complexity is epitomized by the functional duality of Lipocalin-2 (LCN2) [65,66]. While classically characterized as a siderophore scavenger that limits bacterial growth, Hyeong S. et al. reveal a pathological inversion during sterile lung injury (e.g., LPS-induced) [77]. In the absence of bacterial targets, LCN2 functions aberrantly as a pathogenic iron shuttle, delivering excessive iron into macrophages, thereby fueling Fenton-mediated tissue injury. This dictates a structure-matching therapeutic principle: LCN2 function should be preserved for siderophore-producing infections but inhibited during sterile inflammation.

Furthermore, emerging evidence is reframing our understanding of bactericidal mechanisms: iron is not merely a contested nutrient but a host-deployed biochemical effector. Against intracellular pathogens, macrophages counter-intuitively pump ferrous iron into the bacterial vacuole [78]. This influx serves to generate highly toxic hydroxyl radicals via the Fenton reaction, executing a targeted oxidative attack that induces in situ ferroptosis-like death of the bacteria.

However, pathogens have evolved sophisticated counter-mechanisms. Jia T. et al. elucidate an epigenetic manipulation by Pseudomonas aeruginosa. The bacterium secretes the quorum-sensing molecule PQS, which activates the host methyltransferase CNMT. This leads to the aberrant methylation and stabilization of the host Transferrin Receptor 1 (TFR1), forcing the macrophage into uncontrolled iron uptake and subsequent ferroptosis. The lysis of the host cell releases iron-rich contents that support bacterial proliferation [79]. From host-mediated oxidative killing to pathogen-mediated metabolic hijacking, this interplay underscores the intricate challenges of targeting iron metabolism in pulmonary infections.

### 4.5. Macrophage Iron Dysregulation in Renal Aging and Fibrosis

Aging kidneys are progressively characterized by chronic inflammation and severe interstitial fibrosis, a structural deterioration that compromises renal function [80,81,82,83]. Within this pathological microenvironment, dysregulated macrophage iron metabolism operates not merely as a bystander, but acts as a central upstream driver accelerating this fibrotic remodeling [84]. The aging process is characterized by the pathological hyperactivation of the transcription factor Stat1, which transcriptionally represses the cytosolic iron chaperone Pcbp1 (Poly(rC)-binding protein 1). Physiologically, Pcbp1 acts as a molecular escort, safely delivering reactive cytosolic iron to ferritin or iron-dependent enzymes; its deficiency precipitates an uncontrolled accumulation of “orphan” labile iron, triggering severe oxidative stress [84]. The collapse of intracellular iron homeostasis yields a dual pathological outcome: it precipitates macrophage ferroptosis and concurrently drives the acquisition of a Senescence-Associated Secretory Phenotype (SASP). These metabolically compromised macrophages function as “pro-inflammatory beacons,” releasing profibrotic mediators that act in a paracrine manner to drive Epithelial-Mesenchymal Transition (EMT) in adjacent renal tubular cells. The delineation of this “Stat1-Pcbp1-SASP axis” illustrates how aberrant iron metabolism transcends cellular boundaries, translating immunosenescence into irreversible parenchymal fibrosis.

## 5. Therapeutic Frontiers and Clinical Translation

Traditional strategies have predominantly relied on non-specific systemic iron chelation (e.g., deferoxamine, deferiprone and deferasirox) [85,86]. While efficacious, these approaches often function as “blunt instruments,” failing to discriminate between physiological iron reserves and pathological iron overload, and are frequently plagued by systemic off-target toxicity and immunosuppression.

**Figure 5:**
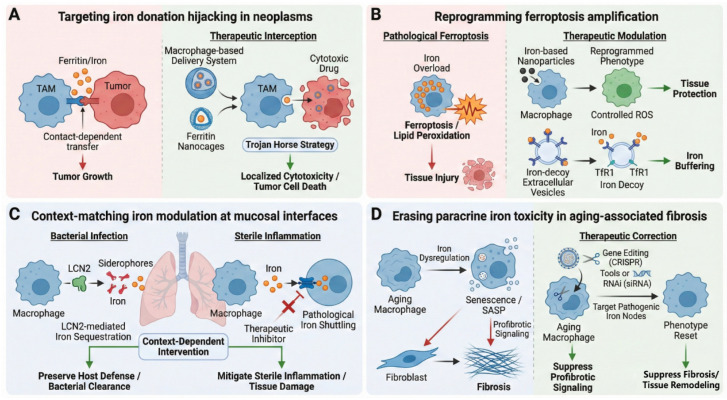
Precision therapeutic strategies targeting macrophage iron dysregulation: Future therapeutic interventions targeting macrophage iron metabolism will shift from non-specific systemic chelation to precise strategies targeting disease-specific hijacking mechanisms. (**A**) In neoplasms, TAM iron donation pathways are hijacked as “Trojan Horse” delivery routes for localized tumor cytotoxicity. (**B**) In cardiovascular and metabolic diseases, ferroptosis amplification is mitigated via nanomedicine-based reprogramming or endogenous iron-decoy vesicles. (**C**) At mucosal interfaces, context-matching modulation preserves host defense against pathogens while mitigating sterile inflammation. (**D**) In aging-associated fibrosis, targeted gene editing or RNAi erases pathogenic iron-handling nodes to suppress paracrine profibrotic signaling.

In contrast, the next generation of therapeutic strategies aims for “surgical precision”: shifting towards interventions that specifically target distinct pathological cell subpopulations and discrete subcellular molecular checkpoints (Figure 5). While these mechanistic breakthroughs offer a compelling blueprint for the future, it is crucial to emphasize that the next-generation precision interventions discussed herein—including targeted nanomedicines and macrophage-engineering strategies—are currently prospective. To date, their therapeutic efficacy has predominantly been demonstrated as proof-of-concept in experimental and preclinical models. Extensive rigorous clinical trials are imperative to validate their safety, scalability, and translational viability in human pathologies.

### 5.1. Targeting TRAIN-Mediated Iron Transfer

The elucidation of the TRAIN mechanism (TRAnsfer of Iron-binding proteiN) has inspired the development of bio-engineered delivery systems designed to repurpose this physiological iron-trafficking axis into a precision conduit for antineoplastic agents. A prime example is the Macrophage-Drug Conjugate (MDC), which utilizes H-Ferritin (HFt) nanocages as robust drug carriers. Macrophages efficiently internalize these drug-loaded nanocages via the specific scavenger receptor MSR1, thereby establishing a stable intracellular drug depot [69]. Capitalizing on the intrinsic tropism of macrophages for hypoxic microenvironments, MDCs actively penetrate dense tumor stroma—a formidable barrier for conventional therapeutics and home to the necrotic core. Upon establishing cell–cell contact with neoplastic cells, the activated TRAIN machinery facilitates the direct cytosolic transfer of the ferritin-bound payload, achieving potent localized cytotoxicity. Preclinical evaluations in refractory models, including pancreatic ductal adenocarcinoma and metastatic breast cancer, demonstrate that this cellular delivery system significantly enhances intratumoral drug accumulation while circumventing systemic toxicity. Furthermore, the demonstrated viability of MDCs following cryopreservation supports their potential development as standardized, “off-the-shelf” clinical products.

### 5.2. Iron-Based Nanomedicine for Macrophage Reprogramming

Exploiting the intrinsic immunomodulatory properties of Iron Oxide Nanoparticles (IONPs) has emerged as a pivotal strategy for remodeling the immunosuppressive Tumor Microenvironment (TME). Unlike conventional chemotherapeutics, IONPs demonstrate a unique capacity for “iron-dependent repolarization,” capable of “re-educating” pro-tumorigenic M2-like Tumor-Associated Macrophages (TAMs) into an anti-tumor M1 phenotype [87]. This process typically initiates with targeted internalization (e.g., via mannose ligand modification); upon entering the acidic lysosomal compartment, IONPs degrade to liberate free iron ions. The ensuing Fenton reaction generates a controlled burst of Reactive Oxygen Species (ROS). Crucially, this oxidative shift functions not as a cytotoxic stressor but as a second messenger, activating canonical pro-inflammatory cascades such as NF-κB and MAPK, thereby driving the transcriptional reversion of macrophage polarization.

Furthermore, integrating IONPs with Photodynamic Therapy (PDT) or Sonodynamic Therapy (SDT) establishes a potent synergistic therapeutic modality. In these combinatorial approaches, iron-based nanomaterials serve as sensitizers that amplify direct tumor cytotoxicity while simultaneously inducing Immunogenic Cell Death (ICD) [88,89,90]. The release of Danger-Associated Molecular Patterns (DAMPs) and tumor antigens during ICD, coupled with the presence of reprogrammed M1 macrophages, creates a positive feedback loop [91]. This mechanism bridges local physical ablation with systemic antitumor immune activation, achieving a dual therapeutic efficacy.

### 5.3. Microbiota–Epigenetic Modulation of Macrophage Iron Metabolism

Modulating the gut microbiota and its metabolic byproducts offers a promising non-invasive strategy to address regulatory niches often inaccessible to conventional pharmacology. This intervention via the “Microbiota-Macrophage Axis” extends beyond simple metabolic supplementation to achieve profound epigenetic remodeling.

In the management of Anemia of Chronic Disease (ACD), the short-chain fatty acid Butyrate has emerged as a potent “pharmaconutrient.” Functioning as a natural HDAC inhibitor, butyrate mediates the epigenetic derepression of the ferroportin (FPN) promoter, effectively lifting the inflammatory blockade on iron efflux. This restoration of macrophage iron mobilization offers a novel therapeutic paradigm that integrates a “Nutri-Epigenetic-Metabolic” trinity, particularly beneficial for anemia associated with Inflammatory Bowel Disease (IBD) [63]. Conversely, in the realm of oncology, synthetic biology is transforming probiotics into “living drugs.” Engineered strains (e.g., modified *L. plantarum*) are designed to colonize the tumor microenvironment and selectively trigger the “nutritional immunity” program in infiltrating macrophages. The consequent upregulation of LCN2 sequesters iron within the local niche, imposing a state of “metabolic starvation” on tumor cells [92,93]. This approach elegantly repurposes commensal bacteria as immunomodulators to execute a precise blockade of tumor iron metabolism.

### 5.4. Endogenous Biologics and Genetic Interventions Targeting Iron Metabolism

Occupying the therapeutic niche between whole-cell therapies and small-molecule drugs, cell-free biologics offer a strategy of superior biocompatibility. Researchers have harnessed autologous macrophage-derived Extracellular Vesicles (EVs) to engineer membrane-based iron scavengers [94]. Naturally enriched with a high density of Transferrin Receptor 1 (TfR1), these exosomes function as roving “biomimetic iron sponges,” efficiently sequestering labile iron from the circulation or interstitium. In models of myocardial ischemia-reperfusion injury or systemic iron overload, these endogenous decoys effectively mitigate local iron toxicity while circumventing the adverse hemodynamic and renal side effects frequently associated with synthetic chemical chelators [74]. In parallel to these scavenging approaches, gene editing and RNA interference (RNAi) technologies are forging a frontier for the “molecular erasure” of pathogenic iron nodes. To dismantle the avid iron-storage phenotype of M2-like Tumor-Associated Macrophages (TAMs), novel hybrid engineered vesicles—fusing bacterial outer membranes with liposomes—have been developed to deliver siRNA specifically against FTH1 [95]. This targeted silencing precisely disables the macrophage’s iron sequestration machinery. The consequently liberated iron triggers ferroptosis or phenotypic reprogramming via the Fenton reaction, thereby reinvigorating the anti-tumor immune microenvironment at the genetic level.

## 6. Conclusions and Perspectives

From the atomic resolution of transporter cryo-EM structures to the systemic complexity of the tumor microenvironment, our understanding of macrophage iron metabolism has undergone a qualitative leap. The accumulated evidence delineates a definitive paradigm shift: the macrophage is not merely a passive “porter” of elemental iron, but an intelligent quantitative modulator of systemic homeostasis. This cellular machinery exhibits extraordinary multidimensional sensing capabilities: transducing mechanical forces via PIEZO1, coupling bioenergetics through the SIRT3-mitochondrial axis, monitoring heme toxicity via Bach1, and encoding environmental stress through epigenetic memory. This integrative capacity empowers the macrophage to translate microenvironmental iron cues into decisive instructions that determine tissue fate—balancing regeneration against fibrosis, and immunity against tolerance.

These mechanistic breakthroughs are redrawing the blueprint for clinical intervention. Strategies for treating anemia are evolving from simple “exogenous supplementation” to using gut microbial metabolites (e.g., butyrate) to “unlock” endogenous iron stores sequestered by inflammation. Conversely, the frontier of oncology is shifting from targeting cancer cells directly to “hijacking” or “reprogramming” the macrophage compartment: exploiting the TRAIN mechanism to deploy drug-loaded macrophages as vectors to penetrate the hypoxic tumor core, or utilizing nanomedicine-induced Fenton reactions to forcibly revert the immunosuppressive M2 phenotype.

Nevertheless, translating the precise modulation of macrophage iron metabolism into clinical reality requires bridging several critical knowledge gaps. Future research must prioritize identifying the elusive molecular entities responsible for direct iron sensing within the macrophage. Furthermore, leveraging spatial transcriptomics to decipher the intrinsic metabolic heterogeneity across different tissue microenvironments—distinguishing, for instance, the distinct regulatory signatures of microglia from alveolar macrophages—will be essential for achieving organ-specific targeting. As we unravel these complexities, the precise tuning of the macrophage as a “Cellular Iron Quantitative Modulator” will undoubtedly serve as a master key to unlocking new therapeutic eras for inflammatory, metabolic, and malignant diseases.

## Data Availability

Data sharing is not applicable to this article as no new data were created or analyzed in this review study.
